# Enlargement of a Preexisting Pulmonary Nodule Mimicking Metastasis during Neoadjuvant Chemotherapy with Pembrolizumab for Triple-Negative Breast Cancer

**DOI:** 10.70352/scrj.cr.26-0267

**Published:** 2026-07-07

**Authors:** Michiko Yamazaki, Rikiya Nakamura, Shouko Hayama, Hideyuki Yamada

**Affiliations:** Division of Breast Surgery, Chiba Cancer Center, Chiba, Chiba, Japan

**Keywords:** triple-negative breast neoplasms, pembrolizumab, immune checkpoint inhibitors, neoadjuvant therapy, pulmonary nodules, pseudoprogression, sarcoid-like reaction, differential diagnosis

## Abstract

**INTRODUCTION:**

Neoadjuvant immunochemotherapy with immune checkpoint inhibitors (ICIs) can yield atypical radiological patterns that may complicate response assessment and management decisions in curative-intent settings.

**CASE PRESENTATION:**

A 50-year-old female with triple-negative breast cancer received neoadjuvant chemotherapy with pembrolizumab. While the primary breast tumor showed marked regression, a preexisting subpleural pulmonary nodule gradually enlarged over 6 months without respiratory symptoms or fluorodeoxyglucose uptake on PET-CT. As metastatic disease could not be excluded based on imaging alone, video-assisted thoracoscopic resection was performed. Histopathology revealed lymphocyte-predominant inflammation with hemosiderin-laden macrophages and no histological evidence of malignancy, consistent with a benign inflammatory lesion. The final pathological diagnosis was inflammatory change arising within a preexisting pulmonary bulla. The patient showed no signs of recurrence on follow-up imaging performed 1 year after breast surgery.

**CONCLUSIONS:**

Discordant enlargement of a solitary pulmonary nodule during ICI-based therapy requires serial imaging and awareness of PET limitations for small nodules. When feasible, tissue confirmation may prevent premature abandonment of curative-intent treatment.

## Abbreviations

AUC
: area under the curve
CA15-3
: cancer antigen 15-3
CEA
: carcinoembryonic antigen
ER
: estrogen receptor
FDG
: fluorodeoxyglucose
HER2
: human epidermal growth factor receptor 2
ICI
: immune checkpoint inhibitor
iRECIST
: immune Response Evaluation Criteria in Solid Tumors
PgR
: progesterone receptor
SUVmax
: maximum standardized uptake value
TNBC
: triple-negative breast cancer
VATS
: video-assisted thoracoscopic surgery

## INTRODUCTION

The KEYNOTE-522 trial demonstrated the efficacy of neoadjuvant chemotherapy combined with pembrolizumab, followed by adjuvant pembrolizumab monotherapy, in patients with high-risk early-stage TNBC.^1)^ As a result, this treatment strategy is increasingly adopted in clinical practice.

Neoadjuvant treatment requires systematic monitoring of tumor shrinkage and new lesion development. Objective assessment of tumor burden commonly relies on standardized criteria, including World Health Organization and Response Evaluation Criteria in Solid Tumors guidelines.^2)^ However, responses to ICIs differ from those to conventional cytotoxic agents or molecular-targeted therapies and may produce atypical radiologic patterns.^3)^ Transient tumor enlargement and new inflammatory lesions, for example, can mimic progressive disease and complicate clinical decision-making when conventional response criteria are applied.

To better account for immune-related response patterns, the iRECIST guidelines incorporate follow-up imaging to distinguish true progression from immune-related response patterns, such as pseudoprogression.^4)^

We present a case of TNBC in which a pulmonary lesion enlarged during neoadjuvant chemoimmunotherapy, raising suspicion of metastatic disease. We outline the clinical course, radiological features, and histopathological findings to highlight relevant diagnostic considerations.

## CASE PRESENTATION

A 50-year-old female presented with a palpable mass in her right breast in May of Year X. The patient had no significant medical history and was not taking any regular medications. The family history included maternal lung and colorectal cancers. Blood tests at the initial presentation showed normal CEA and mildly elevated CA15-3 levels (4.3 ng/mL and 31.3 U/mL, respectively).

Breast ultrasonography revealed a mass measuring approximately 65 mm in the upper outer quadrant of the right breast. Core needle biopsy confirmed an invasive ductal carcinoma of histological grade III. Immunohistochemical analysis demonstrated ER negativity, PgR negativity, and an HER2 score of 1+, consistent with TNBC. Initial staging workup, including chest CT and bone scintigraphy, showed no definitive evidence of distant metastasis. Although a small solitary pulmonary nodule was noted in the left lower lobe, it was not considered diagnostic of pulmonary metastasis at that time because of its small size and the absence of other metastatic lesions. Therefore, the patient was considered to have resectable TNBC and was treated with curative intent. Based on the pretreatment findings, the patient was clinically staged as cT3N1M0, Stage IIIA, according to the Union for International Cancer Control (UICC) TNM classification.

**Fig. 1:**
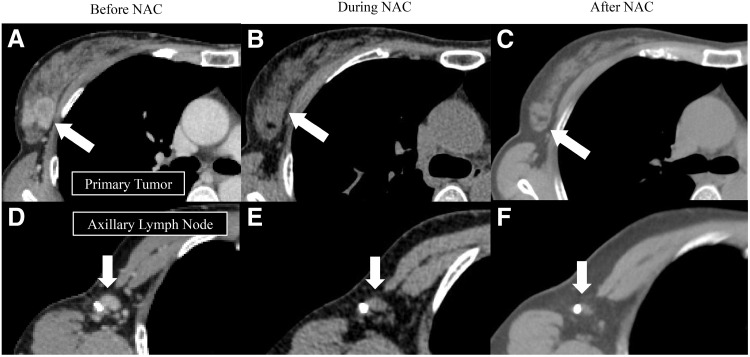
Serial CT images of the primary tumor and axillary lymph node before and after neoadjuvant therapy. (**A**–**C**) Axial CT images of the primary tumor in the right breast demonstrating gradual tumor shrinkage in response to ICI-based neoadjuvant chemotherapy. (**A**) Image from May of Year X (before treatment initiation), showing tumor diameter of 65 mm. (**B**) Image from August of Year X, after 4 cycles of pembrolizumab (200 mg every 3 weeks) combined with weekly paclitaxel (80 mg/m^2^) and carboplatin (AUC 1.5). (**C**) Image from December of Year X, after 4 additional cycles of pembrolizumab with epirubicin (90 mg/m^2^) and cyclophosphamide (600 mg/m^2^), showing a decrease in tumor diameter to 30 mm. (**D**–**F**) CT images of ipsilateral axillary lymph nodes. Images were captured at the same time points as those in (**A**–**C**). A progressive reduction in lymph node size was observed, indicating treatment efficacy. Notably, the lymph node was confirmed to harbor metastasis by fine-needle aspiration cytology before treatment, and a clip was placed in the node for identification. Arrows indicate the primary breast tumor in panels **A**–**C** and the ipsilateral axillary lymph node in panels **D**–**F**. AUC, area under the curve; ICI, immune checkpoint inhibitor; NAC, neoadjuvant chemotherapy

In June of Year X, neoadjuvant chemotherapy was initiated in accordance with the KEYNOTE-522 regimen. The patient received 4 cycles of pembrolizumab (200 mg every 3 weeks) in combination with weekly paclitaxel (80 mg/m^2^) and carboplatin (AUC 1.5), followed by 4 cycles of pembrolizumab with epirubicin (90 mg/m^2^) and cyclophosphamide (600 mg/m^2^). After completion of neoadjuvant therapy, the breast tumor had decreased in size to 30 mm, with accompanying marked reduction in axillary lymph node size. Although the axillary lymph nodes had markedly decreased in size, residual nodal involvement was still suspected clinically. In addition, fine-needle aspiration cytology of the axillary lymph node, which had been clipped after confirmation of metastasis before treatment, demonstrated malignant cells after neoadjuvant therapy. Therefore, the posttreatment clinical stage was assessed as ycT2N1M0, Stage IIB, according to the UICC TNM classification (**Fig. 1**).

**Fig. 2:**
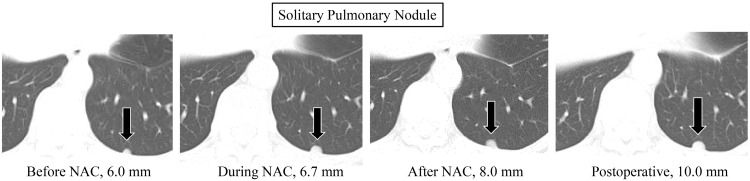
Serial CT images of a solitary pulmonary nodule in the left lower lobe (lung window). The nodule measured 6.0 mm before neoadjuvant chemotherapy (May of Year X), 6.7 mm during neoadjuvant chemotherapy (July of Year X), 8.0 mm after neoadjuvant chemotherapy (December of Year X), and 10.0 mm postoperatively (April of Year X + 1). Arrows indicate the solitary pulmonary nodule in the left lower lobe. NAC, neoadjuvant chemotherapy

**Fig. 3:**
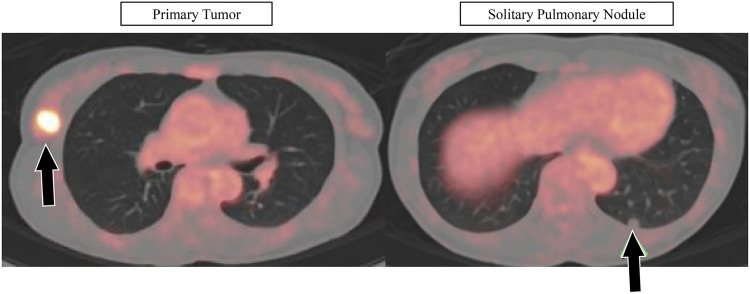
PET-CT findings of the primary tumor and the solitary pulmonary nodule after neoadjuvant chemotherapy (December of Year X). (Left) Image showing strong FDG uptake in the primary breast tumor. (Right) Image of the solitary pulmonary nodule in the left lower lobe showing no abnormal FDG uptake (SUVmax <2.5). Arrows indicate the primary breast tumor on the left image and the location of the solitary pulmonary nodule in the left lower lobe on the right image. FDG, fluorodeoxyglucose; SUVmax, maximum standardized uptake value

At the initial staging workup in May of Year X, chest CT revealed a 6.0-mm round subpleural nodule in the left lower lobe. Given its small size and subpleural location, the differential diagnosis included both benign and malignant etiologies, such as an intrapulmonary lymph node, inflammatory change, metastatic breast cancer, and primary lung cancer. Follow-up CT in July of Year X showed a slight increase in size to 6.7 mm, which was considered within the margin of measurement error, although the lesion was marked for close monitoring in future follow-up imaging. During neoadjuvant chemotherapy, in contrast to the shrinking breast tumor, the size of the pulmonary nodule gradually increased (**Fig. 2**). In December of Year X, PET-CT was additionally performed to evaluate potential metastasis. The lesion did not show abnormal FDG uptake, with an SUVmax of <2.5 (**Fig. 3**). Based on these findings, the likelihood of an FDG-avid malignant lesion was considered low. However, FDG-PET-CT has limited sensitivity for small (<10 mm) pulmonary nodules owing to partial volume effects; therefore, metastatic disease could not be excluded based on PET findings alone.

In January of Year X + 1, the patient underwent a total mastectomy and axillary lymph node dissection. Pathological examination revealed ypT2N2aM0, Stage IIIA, according to the UICC TNM classification, with 4 of 19 lymph nodes positive, histological grade III, the presence of lymphatic invasion, the absence of vascular invasion, ER negativity, PgR negativity, and an HER2 score of 1+.

From March to April of Year X + 1, the patient received postoperative radiotherapy (50 Gy) administered to the regional lymph nodes.

**Fig. 4:**
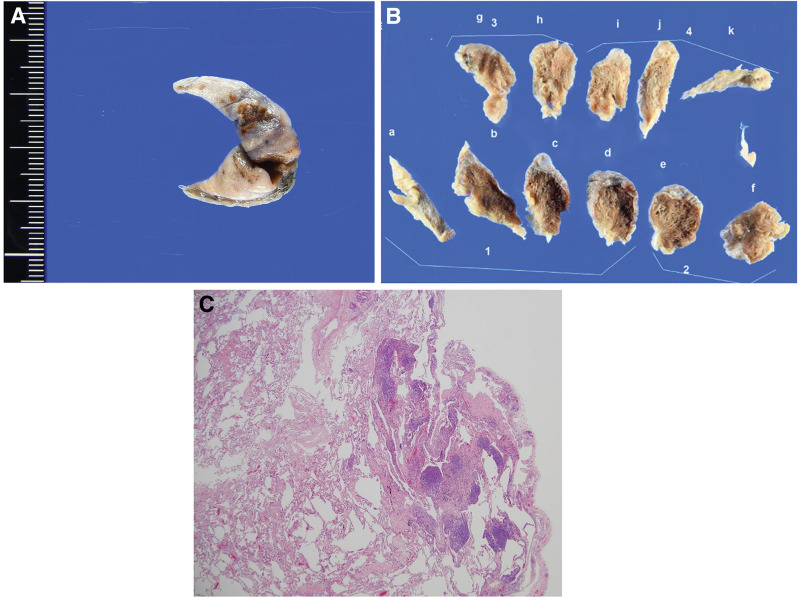
Gross and histopathological findings of the resected pulmonary specimen. (**A**) Resected lung specimen containing a solitary pulmonary nodule. (**B**) Serial sectioning revealed no distinct nodular lesion. (**C**) Histopathological examination demonstrated infiltration of small lymphocytes and hemosiderin-laden macrophages within the alveolar walls, with no evidence of malignancy (H&E staining; Original magnification, ×40). H&E, hematoxylin and eosin

In April of Year X + 1, follow-up CT revealed further enlargement of the pulmonary nodule to 10 mm, confirming continued interval growth on serial imaging and raising concern for malignancy. CT-guided percutaneous biopsy is less invasive than VATS and may be considered for the diagnosis of pulmonary nodules. However, in the present case, the pulmonary nodule was small and solitary, and a negative or nondiagnostic biopsy result would not have definitively excluded malignancy. Because neither metastatic breast cancer nor primary lung cancer could be excluded and the lesion was surgically resectable, VATS with partial lung resection was performed in May of Year X + 1 to establish a definitive diagnosis. Histopathological examination showed infiltration of small lymphocytes and hemosiderin-laden macrophages into the alveolar walls, with no evidence of malignancy. No epithelioid granulomas were observed, and both Ziehl–Neelsen and Periodic Acid-Schiff staining were negative. The final pathological diagnosis of the lung lesion was an inflammatory change arising within a preexisting pulmonary bulla. No histological evidence of breast cancer metastasis or primary lung cancer was identified (**Fig. 4**). The patient showed no signs of recurrence on follow-up imaging performed 1 year after breast surgery.

## DISCUSSION

In this case, a small pulmonary nodule enlarged during neoadjuvant chemoimmunotherapy for TNBC, whereas the primary breast tumor showed a favorable response, making differentiation from pulmonary metastasis of breast cancer challenging. Pathological evaluation by VATS ultimately revealed an inflammatory change, confirming that the pulmonary lesion was not metastatic. This case highlights the need to avoid prematurely classifying small indeterminate pulmonary lesions detected at the initial diagnosis of breast cancer as stage IV disease, and to interpret changes in pulmonary lesions cautiously during treatment with ICIs, taking into account pseudoprogression or pseudoprogression-like immune-related inflammatory changes.

In recent years, the interpretation of small pulmonary lesions in breast cancer has become increasingly complex, partly because the wider use of CT and PET-CT has increased the detection of small pulmonary nodules at the time of initial diagnosis. However, it is often difficult to determine whether such small lesions are benign or malignant based solely on morphological features or FDG uptake. Differential diagnoses include pulmonary metastasis from breast cancer, inflammatory change, post-infectious change, granulomatous disease, primary lung cancer, and tumor-like inflammatory lesions. Therefore, when a small pulmonary lesion is detected at the initial diagnosis of breast cancer, distant metastasis should not be diagnosed solely on this basis, as doing so may deprive the patient of the opportunity for curative-intent treatment.^5)^

The introduction of ICIs has further complicated this diagnostic process, because pseudoprogression and immune-related inflammatory changes have been reported during treatment with these agents.^3)^ Reports of pseudoprogression in breast cancer remain limited, although a case report has described pseudoprogression during atezolizumab monotherapy for metastatic TNBC.^6)^ In conventional oncologic practice, an enlarging pulmonary nodule during systemic therapy is often interpreted as a metastatic lesion. However, during treatment with ICIs, a lesion may appear to enlarge transiently because of immune cell infiltration or localized inflammatory responses rather than true tumor progression.^7)^ In the lung, immune-related inflammatory changes, including organizing pneumonia-like changes, granulomatous reactions, and localized inflammatory nodules, may also mimic metastatic pulmonary nodules on imaging.^8–10)^

A key issue in the present case was that the therapeutic intent would differ substantially depending on whether the pulmonary lesion represented metastasis. In patients with potentially curable stage II–III TNBC, neoadjuvant chemotherapy with pembrolizumab, followed by adjuvant pembrolizumab, is an important treatment strategy. The KEYNOTE-522 trial demonstrated improvements in pathological complete response and event-free survival, which were the primary endpoints of the trial. More recently, an overall survival benefit has also been reported, further supporting the clinical significance of incorporating ICIs into curative-intent treatment for early-stage TNBC.^1)^ In contrast, if the disease is classified as stage IV, the treatment intent generally shifts from curative treatment to disease control, and the role of surgery differs substantially. Therefore, classifying a patient as having stage IV disease based solely on an indeterminate small pulmonary lesion may lead not only to overdiagnosis but also to undertreatment of a potentially curable disease.^5)^ In the present case, neoadjuvant chemoimmunotherapy was initiated for potentially curable TNBC because the pulmonary lesion was indeterminate, no other organ metastases were detected, and curative potential remained.

When the pulmonary nodule enlarged during neoadjuvant therapy, pulmonary metastasis from breast cancer had to be strongly considered. However, in the present case, the primary breast tumor showed a favorable response, whereas only the pulmonary lesion enlarged, resulting in a discordant response pattern. In particular, during treatment with ICIs, pseudoprogression and several inflammatory tumor-like conditions, including organizing pneumonia-like changes, granulomatous reactions, and localized immune-related pulmonary inflammatory lesions, may mimic metastatic pulmonary nodules on imaging.^6–10)^ Therefore, it was difficult to conclude that the enlargement of the pulmonary lesion alone represented treatment-resistant metastatic disease.

For this reason, although enlargement of the pulmonary lesion was observed, we considered it inappropriate to discontinue neoadjuvant therapy or treat the patient as having stage IV disease on this basis alone. Because the primary tumor was responding to treatment and curative potential remained, treatment was continued with curative intent. Accordingly, the pulmonary lesion was carefully monitored, and curative surgery for the primary breast tumor was performed first.

After breast surgery, the decision to perform VATS for diagnostic confirmation was made based on the pathological response of the primary breast tumor and the subsequent course of the pulmonary lesion. The purpose of VATS was not merely to resect the pulmonary nodule, but to obtain a definitive diagnosis that would guide postoperative treatment planning. If the pulmonary lesion had represented breast cancer metastasis, postoperative treatment would have needed to be restructured as treatment for metastatic breast cancer. In contrast, if the lesion was benign or inflammatory, curative-intent postoperative treatment could be continued. Thus, pathological confirmation was clinically important because the diagnosis of the pulmonary lesion directly affected the therapeutic intent of postoperative management.

Furthermore, even if the pulmonary lesion had represented breast cancer metastasis, the possibility of oligometastatic disease was considered because the lesion was solitary and resectable, with no evidence of metastasis to other organs. Although local therapy for oligometastatic breast cancer is not an established standard treatment, it may be considered in selected patients with the aim of achieving long-term disease control or improving prognosis.^11)^ Therefore, in the present case, VATS was a reasonable option not only for diagnostic confirmation but also as a procedure that could have provided local control if the lesion had been metastatic.

The final pathological diagnosis of the pulmonary lesion was an inflammatory change, and pulmonary metastasis from breast cancer was excluded. This result supported the decision not to classify the patient as having stage IV disease based solely on the small pulmonary lesion detected at initial diagnosis or its enlargement during treatment. It also demonstrated that, during treatment including ICIs, enlargement of a pulmonary lesion on imaging does not necessarily indicate tumor progression. In particular, when the treatment response differs between the primary breast tumor and a pulmonary lesion, conditions other than metastasis, including pseudoprogression or pseudoprogression-like inflammatory changes, should be actively considered.

The key lesson from this case is that small pulmonary lesions in patients with TNBC should not be regarded as evidence of stage IV disease based on imaging findings alone. Instead, treatment response, longitudinal changes in the lesion, and the possibility of immunotherapy-related inflammatory changes should be comprehensively evaluated. In the era of immunotherapy, simple interpretation of an enlarging small pulmonary lesion as metastatic progression may be misleading because of the possible presence of pseudoprogression or tumor-like inflammatory changes. When the diagnosis of a pulmonary lesion directly affects decisions regarding continuation of curative-intent treatment, selection of postoperative adjuvant therapy, or transition to treatment for metastatic breast cancer, pathological confirmation, including VATS, should be considered.

This report has several limitations. First, this is a single case report, and the findings may not be generalizable. Second, although histopathology excluded malignancy and supported a benign inflammatory process, a definitive causal link to pembrolizumab could not be established. Third, FDG-PET has reduced sensitivity for subcentimeter nodules because of partial volume effects, underscoring the value of multimodal assessment and tissue confirmation when management decisions may change. Finally, longer follow-up and additional cases are required to better characterize this imaging pattern and refine practical decision-making strategies in the curative-intent setting.

## CONCLUSIONS

This case report describes a patient with TNBC in whom a preexisting solitary pulmonary nodule enlarged during pembrolizumab-based neoadjuvant chemotherapy, mimicking metastatic progression on imaging.

Video-assisted thoracoscopic resection revealed no malignancy. These findings were most consistent with a benign inflammatory change, possibly immune-related, in the context of ICI therapy. This case highlights that discordant pulmonary findings during immunochemotherapy should be interpreted cautiously and that a stepwise approach incorporating the limited sensitivity of FDG-PET for small nodules, serial imaging, and tissue confirmation, when feasible, may support appropriate continuation of curative-intent treatment.

## DECLARATIONS

### Use of artificial intelligence tools

During the preparation of this study, AI-assisted tools (ChatGPT and OpenAI) were used to improve the readability and language of the manuscript. All content was subsequently reviewed and edited by the authors, who take full responsibility for the final version.

### Availability of data and materials

The data generated in this study are included in this article and its figures.
